# The Rationale for Postoperative MRI Surveillance in Lobular Breast Cancer

**DOI:** 10.3390/cancers18050776

**Published:** 2026-02-28

**Authors:** Lora Grbanović, Lucija Kovačević, Ana Smolić, Maja Prutki

**Affiliations:** 1 Department of Radiology, University Hospital Centre Zagreb, Kišpatićeva 12, 10000 Zagreb, Croatia; 2 Health Care Center Zagreb–West, Prilaz Baruna Filipovića 11, 10000 Zagreb, Croatia; 3 School of Medicine, University of Zagreb, Šalata 3, 10000 Zagreb, Croatia

**Keywords:** breast carcinoma, lobular carcinoma, magnetic resonance imaging, recurrence

## Abstract

**Background/Objectives:** Postoperative breast cancer imaging follow-up primarily relies on annual mammography, while magnetic resonance imaging is reserved for selected cases. Invasive lobular carcinoma has growth characteristics that can limit detection on conventional imaging and may influence recurrence patterns. This study aimed to determine whether histological type can help guide postoperative magnetic resonance imaging follow-up. Secondary objectives were to assess inter-reader agreement and evaluate timing of new breast malignancies in relation to tumor features. **Methods:** This retrospective, single-center study included patients with invasive breast carcinoma diagnosed during 2015 and 2016 who underwent surgery, had pre-treatment magnetic resonance imaging, at least one postoperative magnetic resonance imaging within five years, and a minimum follow-up of seven years. Clinical, pathological, and imaging data were collected. New breast malignancies included both local recurrences and second primary tumors. Two radiologists independently reviewed postoperative magnetic resonance imaging examinations in patients, and inter-reader agreement was assessed using Cohen’s kappa coefficient. **Results:** Seventy-seven patients with 80 tumors were included. Six patients (8%) developed a new breast malignancy. New malignancies were more common in patients with invasive lobular carcinoma (*p* = 0.04). Magnetic resonance imaging detected all new malignancies in patients with primary invasive lobular carcinoma, whereas mammography missed two of three cases. New breast malignancies were classified as early (within 5 years of diagnosis and initial treatment) or late (at or after 5 years); most occurred within five years, with one late occurrence at 5.6 years. Inter-reader agreement for both magnetic resonance imaging and mammographic detection showed complete concordance (κ = 1.00). **Conclusions:** Patients with primary invasive lobular carcinoma had a higher rate of new breast malignancies, and postoperative magnetic resonance imaging detected new malignancies missed by mammography. These findings suggest that tumor histology may be relevant when considering postoperative imaging and that patients with invasive lobular carcinoma may benefit from magnetic resonance imaging follow-up.

## Simple Summary

Mammography remains the primary method for postoperative follow-up in breast cancer, despite its known limitations in detecting certain tumor subtypes. Invasive lobular carcinoma is a common type of breast cancer that often grows without forming a distinct mass, making recurrences more difficult to detect on standard imaging. This study evaluated whether magnetic resonance imaging can more effectively detect new breast malignancies after surgery, particularly in patients with invasive lobular carcinoma. In this retrospective study, new breast malignancies were more frequent in patients with invasive lobular carcinoma and were more often detected by magnetic resonance imaging than by mammography. These findings suggest that magnetic resonance imaging may provide added value in the follow-up of patients with invasive lobular carcinoma and may help improve detection of new breast malignancies post-surgery.

## 1. Introduction

Breast cancer is the most commonly diagnosed cancer among women worldwide [1].

Imaging plays a central role in the detection and staging of breast cancer, as well as in treatment planning and follow-up [2]. The Breast Imaging Reporting and Data System (BI-RADS) has been widely adopted to ensure consistent radiological reporting and guide further management [3].

Breast cancer is a heterogeneous disease with varying morphology, histology, molecular subtype, therapeutic response, recurrence risk and prognosis [4]. Invasive lobular carcinoma (ILC) accounts for up to 15% of breast cancers, making it the second most prevalent histologic type after invasive breast carcinoma of no special type (NST; previously known as invasive ductal carcinoma) [5,6]. ILC tends to present with larger size, lower histologic grade, higher rates of estrogen receptor (ER) and progesterone receptor (PR) positivity, more frequent HER2 negativity, lower Ki-67 expression and a stronger association with the luminal A molecular subtype [7,8]. Functional inactivation of E-cadherin disrupts cell adhesion and results in an infiltrative growth pattern in ILC, which has important implications for detection on imaging and achieving negative surgical margins [9,10].

ILC presents a diagnostic challenge both radiologically and clinically, as it often does not form a palpable mass [10]. On a macroscopic level, as assessed by radiologic imaging and large-format, thick-section histopathology, this breast cancer subtype largely preserves the normal architecture of the breast, with marked architectural distortion occurring only in advanced stages due to the extensive deposition of fibrous tissue, typically without formation of a discrete mass and instead presenting as indistinct thickening and progressive shrinking of the breast [11,12]. Furthermore, earlier studies suggest that classic ILC is not a uniform entity and can present mammographically either as extensive architectural distortion without a central mass or as a small stellate or circular mass, with significant implications for long-term survival: patients with ILC presenting as a mass had markedly better long-term survival (84% vs. 56% for tumors presenting as architectural distortion) [13].

Conventional mammography and ultrasound frequently underestimate tumor size, multifocality, and multicentricity in ILC, leading to positive surgical margins in up to two thirds of patients undergoing breast-conserving surgery [14,15]. The limitations of these imaging modalities also persist in the postoperative setting, as scar tissue can mimic recurrence [16].

Magnetic resonance imaging (MRI) is the most sensitive imaging modality for the detection of breast cancer, with reported sensitivities ranging from 85% to 100% [17]. Preoperatively, it more accurately evaluates local tumor extent, multifocality, multicentricity, and contralateral disease than conventional imaging, detecting additional lesions that can alter surgical management in up to 62% of patients [18,19,20]. However, its routine preoperative use remains controversial, and most guidelines support selective use, particularly for ILC.

Postoperative contrast-enhanced MRI demonstrates higher sensitivity than conventional modalities for detecting recurrent and second breast tumors, as well as distinguishing scar tissue from malignancy [21,22]. However, recommendations differ across guidelines, and the role of MRI in postoperative follow-up remains unclear [23]. Current post-treatment imaging guidelines mostly rely on annual mammography, while routine use of ultrasound or MRI is generally not recommended [24,25]. When indicated, MRI is reserved for high-risk patients, although definitions of high risk vary between guidelines. It is also used in specific scenarios, such as when the initial breast cancer is not detectable on other imaging or to differentiate scar tissue from recurrence. Although some guidelines recommend preoperative MRI for ILC to assess tumor size for surgical planning, none of the major guidelines formally include ILC as an independent indication for routine postoperative MRI [25].

The timing of breast cancer recurrence is influenced by multiple factors, the most significant being hormone receptor status [6]. Patients with ER-positive tumors, including most ILCs, face a higher risk of late recurrence after five years [8,26,27]. In contrast, a higher Ki-67 index is associated with early recurrence [28]. Regarding molecular subtypes, luminal A tumors show a slow, sustained risk, luminal B tumors relapse mostly within five years, HER2-enriched tumors display early and late peaks influenced by Ki-67 and triple-negative tumors exhibit an early peak when proliferation is high [29].

Despite the recognized advantages of MRI and the distinct characteristics of ILC growth and recurrence patterns, the value of postoperative MRI remains uncertain, and it is unclear whether histologic type should influence follow-up imaging.

The primary aim of this study was to explore the potential role of histologic type in guiding MRI follow-up after breast cancer surgery, with a focus on lobular carcinoma, by examining radiological, anatomical, and histological characteristics of new breast malignancies. Secondary aims were to evaluate inter-reader agreement and assess new malignancy timing in relation to ER, PR, HER2, Ki-67, and molecular subtype.

## 2. Materials and Methods

This retrospective, single-center study was approved by the Ethics Committee of the University Hospital Centre Zagreb and the Ethics Committee of the University of Zagreb School of Medicine. Informed consent was waived due to the retrospective design.

**Figure 1:**
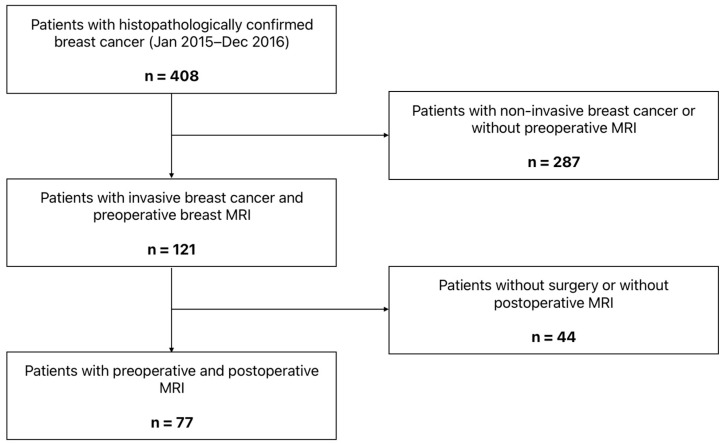
Flowchart of patient selection.

Patients diagnosed with invasive breast cancer by core needle biopsy between 1 January 2015, and 31 December 2016, were identified through the hospital information system. Only patients with available preoperative breast MRI were included. Exclusion criteria were absence of surgical treatment, lack of MRI within five years after surgery, and follow-up of less than seven years. The patient selection process is shown in Figure 1.

Data were extracted from medical records, including patient age at diagnosis, tumor diameter and laterality, histological type of the primary and new tumors, expression of ER, PR, HER2, Ki-67 and breast cancer subtypes (surrogate subtypes) determined by immunohistochemistry of the primary tumors, nodal status and clinical follow-up data. Both local recurrences and second primary tumors were considered new breast malignancies. Histopathological analysis of core biopsy samples served as the reference standard for diagnosing new malignancy.

Suspected lesions on postoperative imaging were classified as true positives if histopathological analysis of biopsy samples confirmed the presence of a breast malignancy. Lesions were considered false positives if histopathology was benign or, in cases where biopsy was not performed, if at least two years of clinical and radiological follow-up showed no evidence of malignancy. True negatives were defined as examinations with no suspicious findings and no subsequent malignancy during follow-up, and false negatives as lesions not initially detected but later confirmed as malignancy during follow-up.

New malignancies were analyzed according to histologic type, receptor status (ER, PR, HER2), Ki-67 index, surrogate subtype and time to new malignancy. New malignancy occurrence was analyzed at the patient level, while tumor characteristics (ER, PR, HER2, Ki-67 and surrogate subtype) were used to describe tumors associated with new malignancies.

All MRI examinations were performed on a 1.5 T MR system (Avanto, Siemens, Erlangen, Germany) using a dedicated breast coil, with patients positioned prone and both hands placed alongside the body. A standardized multiparametric protocol included the following sequences: Axial Turbo Inversion Recovery Magnitude (TIRM) sequence, Axial T2-weighted non-fat-saturated sequence, and Axial 3D T1-weighted fat-saturated sequence acquired before and at five time points after intravenous administration of 0.1 mmol/kg body weight of gadoterate meglumine (Dotarem^®^, Guerbet, Princeton, NJ, USA) into the antecubital vein using an automatic injector at a flow rate of 3.5 mL/s, followed by a 20 mL saline flush. Axial diffusion-weighted imaging (DWI) sequences were performed with b-values of 50, 750, and 1000 s/mm^2^, and an apparent diffusion coefficient (ADC) map was automatically generated on a commercially available workstation.

Two readers, a radiology resident and an experienced breast radiologist, independently reviewed follow-up breast MRI and mammography studies of patients with new breast malignancies. They had access to patient history but were blinded to outcomes. MRI and mammography readings were performed one month apart, with patients who had new malignancies presented in a random order. Each new lesion was categorized according to the BI-RADS classification. BI-RADS assessments were dichotomized, with BI-RADS 4-5 considered positive and BI-RADS 2-3 considered negative for malignancy. Agreement between the two radiologists was assessed using Cohen’s kappa coefficient.

The distribution of continuous variables was assessed using the Shapiro–Wilk test. Normally distributed data were presented as mean and standard deviation (SD). Non-normally distributed data were presented as median and interquartile range (IQR). Subgroup comparisons were performed using the Chi-square or Fisher’s exact test for categorical variables and the *t*-test or Mann–Whitney U test for continuous variables, as appropriate. In cases with low cell counts, the *p*-value was calculated using Monte Carlo simulation. Statistical significance was set at *p* < 0.05. All analyses were performed in Python (version 3.10) [30].

## 3. Results

### 3.1. Patient and Primary Tumor Characteristics

A total of 77 patients with 80 tumors met the inclusion criteria. The mean age at diagnosis was 57 ± 10 years. Histologically, 66 of 80 tumors (83%) were NST, 13 (15%) were ILC, and 2 (2%) were other types. During follow-up, new breast malignancies were observed in six (8%) patients. Characteristics of tumors and patients with and without new malignancies are presented in Table 1. Since the focus was on histologic type, particularly ILC, NST was combined with other tumor types for comparison with ILC. The proportion of ILC was significantly higher in patients with new malignancies (*p* = 0.04). There were no significant differences in age, hormone receptor status, HER2 status, or Ki-67 index.

### 3.2. New Breast Malignancies

Characteristics of primary and new breast tumors are presented in Table 2. Four new malignancies (67%) were ipsilateral and two (33%) were contralateral. The histologic type of new malignancy differed from the primary tumor in three (50%) patients.

### 3.3. Imaging Detection of New Breast Malignancies

All patients underwent mammography within seven months of MRI. Imaging-detected malignancy was defined as a BI-RADS 4 or 5 lesion.

**Figure 2:**
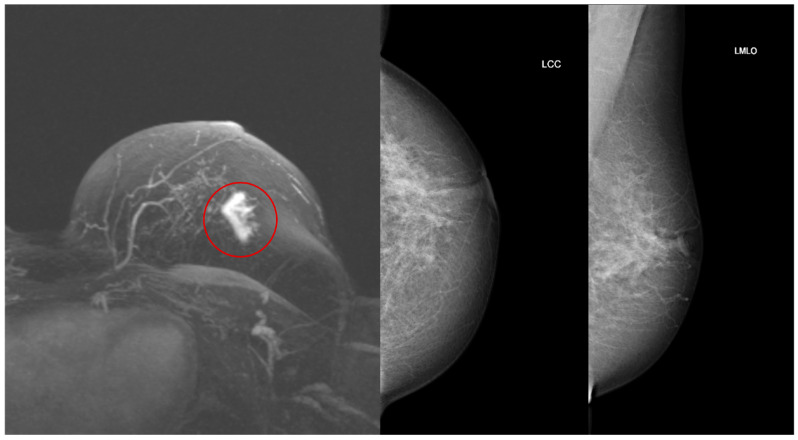
Maximum intensity projection image of breast MRI (**left**), and craniocaudal (LCC) and mediolateral oblique (LMLO) mammograms (**middle** and **right**) in a patient with a new malignancy in the left breast. The mass is visible on MRI (red circle) but not detected on mammography.

**Figure 3:**
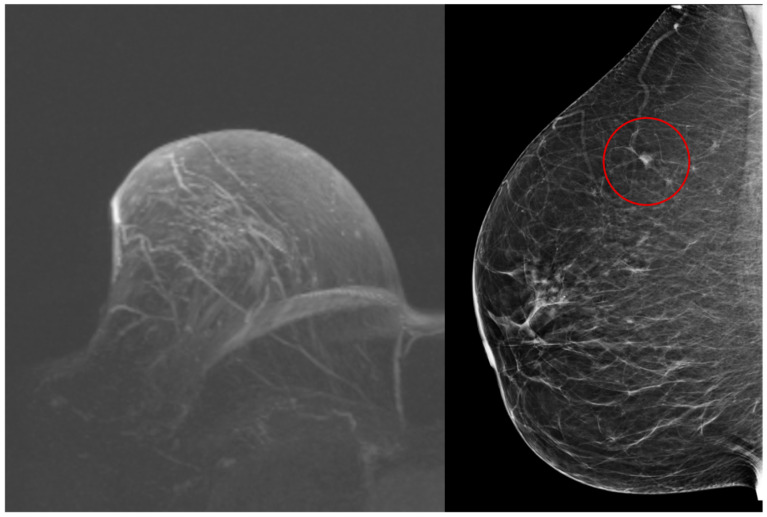
Maximum intensity projection image of breast MRI (**left**) and mediolateral oblique mammogram (**right**) in a patient with a new malignancy in the right breast. The mass is visible on mammography (red circle) but not detected on MRI.

MRI identified both malignancies that were not detected on mammography; an example is shown in Figure 2. One recurrence of contralateral NST carcinoma was detected only by mammography. On mammographic evaluation, the lesion appeared as a mass, while it was not visualized on MRI (Figure 3).

Among patients with primary ILC, two of three new malignancies (66.7%) were identified only on MRI. In contrast, mammography detected all three new malignancies in patients with primary NST carcinoma, while MRI failed to identify one case (33.3%) (Table 2).

Out of 17 suspicious breast lesions detected on postoperative MRI, 11 were ultimately benign and 6 were confirmed as malignant. Among the remaining patients, there were 59 true negatives and one false negative. This corresponds to a false-positive rate of 16% for MRI in detecting new breast malignancies.

### 3.4. Timing of New Breast Malignancy

**Figure 4:**
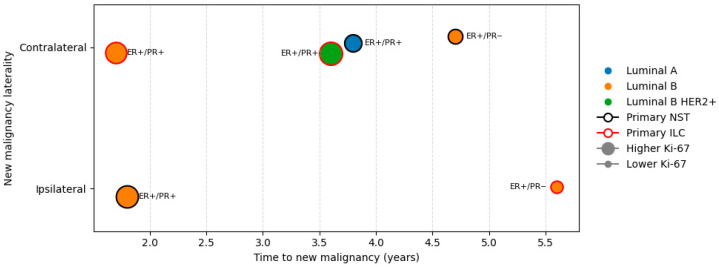
Timeline of new breast malignancies with corresponding primary tumor characteristics. Each dot represents a single tumor. The *y*-axis indicates whether new malignancy occurred in the same or contralateral breast relative to the primary tumor. Dot color represents the molecular subtype of the primary tumor, dot size reflects the Ki-67 index of the primary tumor, and edge color indicates primary tumor histology. ER and PR status of the primary tumor are labeled next to each dot.

Time to new breast malignancy ranged from 1.7 to 5.6 years, with five new malignancies occurring early (less than 5 years) and one occurring late at 5.6 years. Figure 4 shows the timeline of new malignancies and relevant characteristics of the primary tumors.

All primary tumors were ER positive. New malignancies tended to occur later in patients with primary PR-negative tumors, around 5 years after surgery. Both early and late new malignancies were observed among patients with primary luminal B tumors, while new tumors in primary luminal A and luminal B HER2+ occurred at similar times, at 3.8 and 3.6 years, respectively. New malignancies occurred later in primary tumors with lower Ki-67 indices.

A single late new malignancy at 5.6 years was observed in a patient with a primary ILC tumor. The remaining new malignancies occurred within the first five years and arose in patients with primary tumors of both NST and ILC histologic types. To extend the original follow-up, patient outcomes were reviewed for up to 10 years. Some patients were lost to follow-up, one patient died, and others did not meet the 10-year follow-up criterion; however, no additional breast malignancies were identified.

At the time of primary diagnosis, five patients were node-negative and one was node-positive. In node-negative patients, new breast malignancies occurred over a wide time range (1.7–5.6 years), whereas the single node-positive patient experienced new malignancy at 3.8 years. No clear association between patient age or nodal status and time to new malignancy was observed (Table 2).

Although the earliest new malignancy (1.7 years) occurred in the patient with the largest primary tumor (3.8 cm), it arose in the contralateral breast, was of a different histologic type, and almost certainly represented a second primary tumor. For the remaining new malignancies, no clear association between primary tumor size and time to new malignancy was observed (Table 2).

### 3.5. Inter-Reader Agreement

Inter-reader agreement for detecting new breast malignancy on both MRI and mammography showed complete concordance (Cohen’s kappa = 1.00).

## 4. Discussion

This study examined whether the histologic type of breast cancer, particularly invasive lobular carcinoma (ILC), should influence the role of MRI in postoperative follow-up. Our results indicate that, unlike other breast cancer subtypes, invasive lobular carcinoma appears to benefit more from MRI than from mammography in the postoperative follow-up setting. This highlights the potential added value of MRI in detecting new breast malignancies that may be missed by conventional imaging in this specific histologic type.

In this retrospective cohort, postoperative MRI identified all new malignancies in patients with primary ILC, while mammography, the current standard for postoperative follow-up, failed to detect two out of three (66.7%) of these lesions. Although not all new malignancies shared the same histologic type as the primary tumor, these findings further support the superior sensitivity of MRI for breast cancer detection [18,31].

Most new malignancies in patients with primary NST carcinomas were visible on both MRI and mammography. However, MRI failed to detect one new malignancy (33.3%), where a mass visible on mammography was not identified on MRI, possibly due to moderate background parenchymal enhancement (Figure 3). No corresponding mass or non-mass enhancement was observed on MRI.

In contrast to previous reports evaluating MRI detectability in the preoperative setting, where ILC showed a higher proportion of MRI-occult tumors compared with NST carcinoma [32], our findings reflect MRI performance in the postoperative setting. The differing clinical contexts likely account for this discrepancy. Additionally, the low number of new malignancies in our cohort limits the generalizability of these results.

New breast malignancy was defined as the occurrence of a new tumor in either breast. Ipsilateral tumors often share the same histologic characteristics as the primary tumor and in that case are considered true recurrences, while contralateral tumors are generally regarded as new primary tumors [33,34]. Four of six new malignancies in our cohort occurred in the contralateral breast, and three of these (75%) were histologically different from the primary tumor. Both ipsilateral new malignancies shared the same histologic type as the primary tumor and were therefore considered true recurrences—one was ILC and the other NST. This aligns with previous studies, which report similar local recurrence rates between these histologic types [35]. To avoid misclassification of synchronous or multifocal disease as recurrence, only patients with preoperative breast MRI were included to ensure accurate initial disease staging. Patients with primary invasive lobular carcinomas showed a significantly higher proportion of new breast malignancies compared to other histologic types (*p* = 0.04); however, only one of those tumors was also of ILC type and could be considered a true recurrence. Two of three patients with primary ILC developed contralateral tumors of different histology (NST and DCIS), and none of the patients with primary NST developed a new tumor of ILC type.

Most new breast malignancies (83%) occurred within five years of the primary diagnosis, with only one late malignancy observed at 5.6 years in a patient with a primary ILC tumor during the clinical follow-up in our study, which was a true recurrence. This finding aligns with previous reports indicating that ILC can exhibit late recurrences [36]. Although a prior study reported a higher incidence of second breast cancer in ER-negative patients, all primary tumors in patients with new malignancies in our study were ER-positive, which is consistent with the well-established fact that invasive lobular carcinomas are usually hormone-receptor positive [7,37]. The timing of true recurrences aligns with the literature, highlighting a persistent risk of recurrence in ER-positive breast cancer both during and after the first five years of follow-up [26]. In line with previous studies, we observed an inverse relationship between Ki-67 index and time to recurrence [28].

The false-positive rate of 16% observed in our postoperative MRI cohort falls into the range of previously reported rates in screening breast MRI in high-risk women [38]. Furthermore, of the 11 false-positive findings on MRI, only 4 led to biopsy, while the remaining 7 were resolved with subsequent targeted ultrasound, demonstrating that most false-positive MRI findings can be clarified with noninvasive imaging prior to biopsy.

Our findings show that MRI and mammography provide highly reproducible assessments of new malignancies, with high inter-reader agreement (κ = 1) despite differences in reader experience. This highlights the reliability of MRI in the post-treatment setting and supports its use as a complementary tool to standard mammography for detecting post-surgery malignancies. By dichotomizing outcomes based on biopsy recommendation rather than individual BI-RADS categories, our study emphasizes reproducibility for findings that directly impact patient management, increasing the clinical relevance of our results. These data are consistent with previous reports of substantial MRI agreement (κ = 0.91) [39], while extending the evidence by demonstrating reproducibility across readers with varying experience levels.

This study has several limitations. Its retrospective, single-center design and the small number of postoperative breast malignancies limit definitive conclusions. Factors that could influence the frequency or timing of new malignancies, such as type of adjuvant treatment, BRCA mutation status, family history of malignancy and body mass index, were not analyzed. The length of follow-up may not capture all late recurrences. Furthermore, due to the low number of events, this study was not designed to determine the optimal timing, duration, or age-based stratification for postoperative MRI surveillance, and no specific follow-up protocol can be proposed.

## 5. Conclusions

In conclusion, postoperative MRI demonstrated superior detection of new breast malignancies in patients with invasive lobular carcinoma compared with mammography. Given its high reproducibility, MRI may represent a valuable addition to standard imaging follow-up in this subgroup. These findings suggest that tumor histology could be considered when tailoring postoperative imaging strategies, although larger prospective studies are warranted to further define its role.

## Figures and Tables

**Table 1:** Patient (n = 77) and primary tumor characteristics (n = 80) with regard to new malignancy occurrence.

		No New Breast Malignancy	New Breast Malignancy	*p*-Value
**Patient age (years; mean ± SD)**		58 ± 10	54 ± 7	0.30
Histologic type (n; %)	NST + other	63 (85%) + 2 (3%)	3 (50%) + 0 (0%)	0.04
	ILC	9 (12%)	3 (50%)	
ER status (n; %)	+	66 (89%)	6 (100%)	1.00
	-	8 (11%)	0 (0%)	
PR status (n; %)	+	61 (82%)	4 (67%)	0.31
	-	13 (18%)	2 (33%)	
HER2 status (n; %)	+	11 (15%)	1 (17%)	1.00
	-	63 (85%)	5 (83%)	
Ki-67 index (median, IQR)		25 (11–41)	18 (10–23)	0.16
Surrogate subtype * (n; %)	Luminal A	28 (38%)	1 (17%)	0.63
	Luminal B HER2 -	30 (41%)	4 (67%)	
	Luminal B HER2 +	8 (11%)	1 (17%)	
	HER2 +	3 (4%)	0 (0%)	
	Triple negative	5 (7%)	0 (0%)	

* subtypes determined by immunohistochemistry.

**Table 2:** Characteristics of patients with new breast malignancy and their primary and new tumors.

		Patient					
		A	B	C	D	E	F
**Primary** **tumor**	Age at diagnosis (years)	45	53	61	61	59	46
	Diameter (mm)	21	20	15	38	25	15
	Lymph node status	-	-	+	-	-	-
	ER status	+	+	+	+	+	+
	PR status	-	+	+	+	-	+
	HER2 status	-	+	-	-	-	-
	Ki-67	10%	25%	14%	21%	7%	23%
	Surrogate subtype	Luminal B	Luminal B HER2+	Luminal A	Luminal B	Luminal B	Luminal B
	Histology	NST	ILC	NST	ILC	ILC	NST
**New breast** **tumor**	Time to diagnosis (years)	4.7	3.6	3.8	1.7	5.6	1.8
	Histology	NST	NST	DCIS	DCIS	ILC	NST
	Laterality (*)	C	C	C	C	I	I
	Mammographic detection (**)	+	-	+	+	-	+
	MRI detection (**)	-	+	+	+	+	+

* new tumor laterality—contralateral (C), ipsilateral (I); ** interpreted by a board-certified radiologist.

## Data Availability

The data supporting the findings of this study are not publicly available due to sensitivity but are available from the corresponding author upon reasonable request.

## Abbreviations

The following abbreviations are used in this manuscript:

BI-RADS	The Breast Imaging Reporting and Data System
ILC	Invasive Lobular Carcinoma
NST	Invasive breast carcinoma of No Special Type
DCIS	Ductal Carcinoma in Situ
ER	Estrogen Receptor
PR	Progesterone Receptor
HER-2	Human Epidermal growth factor Receptor 2
MRI	Magnetic Resonance Imaging
